# Sleep Quality and Sleep Hygiene Behaviours Among University Students in Qatar

**DOI:** 10.2147/IJGM.S402399

**Published:** 2023-06-13

**Authors:** Raja Mahamade Ali, Monica Zolezzi, Ahmed Awaisu, Yassin Eltorki

**Affiliations:** 1 Department of Clinical Pharmacy and Practice, College of Pharmacy, QU Health, Qatar University, Doha, Qatar; 2 Department of Pharmacy, Mental Health Hospital, Hamad Medical Corporation, Doha, Qatar

**Keywords:** sleep quality, sleep hygiene, university students, Qatar

## Abstract

**Background:**

Insomnia is a highly prevalent health problem, affecting about one-third of the adult population globally. University students are at a high risk for developing insomnia due to the stressful nature of academic life and often unhealthy sleeping habits. The aim of this study was to explore the prevalence of poor sleep quality and investigate sleep hygiene patterns among university students in Qatar.

**Methods:**

A cross-sectional study was conducted among university students using two validated instruments: the Pittsburgh Sleep Quality Index (PSQI) and the Sleep Hygiene Index (SHI). Data were analyzed using descriptive and inferential statistics, including correlation and multivariate regression analyses.

**Results:**

Two thousand and sixty-two students responded to the web-based survey. The mean PSQI score (7.57±3.03) was indicative of poor sleep quality in approximately 70% of the students. Similarly, the mean SHI score (21.79±6.69) was indicative of poor sleep hygiene patterns in 79% of the students. Academic program type, marital status, gender, and sleep hygiene significantly influenced sleep quality. After controlling for all possible covariates in the multiple regression analysis, sleep hygiene remained as the only factor significantly predicting sleep quality. Students with a good sleep hygiene were about four times more likely to have a good sleep quality compared to those with poor sleep hygiene (adjusted OR= 3.66, 95% CI= 2.8–4.8, p <0.001).

**Conclusion:**

Poor sleep quality and inadequate sleep hygiene practices were highly prevalent among university students in Qatar. Sleep hygiene was found to be the only significant predictor of sleep quality such that those adopting healthy sleep hygiene practices were more likely to have better sleep quality. Interventions to raise awareness on the effect of sleep hygiene on sleep quality among university students are needed.

## Introduction

Approximately, 30% of the global adult population is affected by insomnia, of whom around 10–13% experience the condition chronically.^1^,^2^ Insomnia is characterized by the inability to fall asleep or experiencing frequent sleep disruptions and early morning awakenings, with difficulties in returning to sleep, and is associated with significant impairment of daytime functioning, increased rates of motor vehicle accidents, low productivity, work absenteeism, and overall lower performance.^3^,^4^

Insomnia is diagnosed when these symptoms occur three times per week and persist for at least one month.^5^ Sleep quality is a subjective measure which reflects the satisfaction with the sleep experience, including sleep initiation, maintenance, quantity, and feeling of refreshment upon awakening.^6^ Some factors including sleep environment and life stressors can also lead or worsen insomnia.^5^

Evidence from the literature suggests that poor sleep quality is highly prevalent among university students. Results from several epidemiological studies report insomnia prevalence rates ranging between 30% and 60% in this population.^7–18^ Sleep deprivation, difficulty falling asleep, and interrupted sleep patterns are also very commonly reported complaints among university students.^8^,^19–21^

Sleep hygiene, which consists of a combination of behavioral practices and environmental conditions, also affects sleep quality and can contribute to the development of sleep disorders.^22^ Sleep hygiene practices among university students have also been reported to be inadequate, and worse than in other healthy adults.^23^ Some of the negative sleep hygiene practices frequently reported by university students include irregular sleep schedules, daytime naps, consumption of stimulants, and the use of electronic devices around bedtime.^10^,^12^,^20^,^24–28^

Mental health problems among university students are known to influence the high prevalence of insomnia reported in this population, particularly among those experiencing depression and anxiety.^8^,^12^,^16^,^17^ Studies have also shown that stress and worry about academic performance or examinations are significantly associated with poor sleep quality.^12^,^29–31^ Because sleep disturbances often result in excessive daytime sleepiness, it affects the overall concentration and cognitive function required by students.^16^,^25^,^31^,^32^ Studies have also demonstrated that poor sleep quality is associated with worse academic performance among college students.^24^,^33^

There is limited information on the sleep quality and sleep hygiene patterns among higher education students in Qatar, although studies in other populations have been conducted. For example, an insomnia prevalence rate of 5.5% was reported among community-dwelling adults attending primary care services.^34^ Sleep deprivation (<7 hours of sleep) has also been reported to affect more than 50% of the adult population in Qatar.^35^ Considering the adverse consequences that sleep disturbances may have on students’ academic performance, mental health, and productivity, it is important to determine the prevalence of poor sleep quality and associated factors so that appropriate interventions are implemented. Consequently, the aim of this study was to explore the prevalence of poor sleep quality and associated sleep hygiene patterns among university students in Qatar.

## Methods

### Study Design

A cross-sectional study was conducted among university students using two validated instruments: the Pittsburgh Sleep Quality Index (PSQI) and the Sleep Hygiene Index (SHI). The questionnaires were sent online to all students enrolled at Qatar University (QU), the largest public-owned university in the State of Qatar, in the Fall semester of the 2018–19 academic year. The self-administered questionnaires sent aimed to capture the participants’ sleeping patterns and habits over one month period.

### Study Population and Setting

The study population comprised of all registered undergraduate and postgraduate students at QU. In the 2018–2019 academic year, there were 19,738 students across nine different colleges in the university.^36^ The inclusion criteria for this study were students registered in QU at the time of the study, residency in Qatar and literacy in either Arabic or English.

### Sample Size Determination

Considering insomnia prevalence of 30% among university students, the minimum number of participants required for this study – according to the Cochrane formula – was determined to be 325.^13^,^37^ To ensure reaching the minimum sample size required with a response rate of 15%, the survey was required be sent to 2200 students.

### Sampling Technique

Following a proportionate, stratified random sampling technique, the minimum sample size of 325 was not achieved, therefore, the questionnaires were sent to all QU students (N = 19,738).

### Data Collection Procedure

Students were invited to participate in the study through an email in which they were provided with a link to access the online questionnaire with an option of responding in either English or Arabic. The survey was administered using SurveyMonkey^®^ software and the link was available for one month (7th of January to the 7th of February, 2019). Information collected from the surveys were then exported to SPSS^®^ statistical software for analysis.

### Study Instruments

After conducting an extensive literature review of sleep assessment instruments, two of these instruments were selected for use in the study: (1) the Pittsburgh Sleep Quality Index (PSQI) and (2) the Sleep Hygiene Index (SHI).^38^ Additionally, five demographic questions were provided at the beginning of the survey to capture information related to college designation, age, gender, marital status, country of origin and type of sleep aids used at any point in the past.

#### Pittsburgh Sleep Quality Index (PSQI)

The PSQI is a validated and widely used instrument which is available in many languages including English and Arabic.^39^,^40^ This instrument assesses the respondent’s sleeping patterns, the impact of sleep disturbances on daytime functioning, and the use of sleep aids within the previous month. It consists of 19 self-rated questions and five additional questions to be completed by a roommate or a bed partner. The last five questions of the survey, which should be completed by a roommate, were not included in the PSQI score calculation. The PSQI global score ranges from 0 to 21; a score higher than 5 indicates poor sleep quality, while a score of ≤5 reflects good sleep quality.^39^ This cut-off score was associated with a sensitivity of 89.6% and specificity of 86.5% to detect poor sleepers.^39^ The Arabic translation of the PSQI was validated among a sample of university students in Jordan, and was shown to have an internal consistency of 0.65.^40^ In this study, the PSQI’s total score was computed only for respondents who provided complete and valid answers to all of the survey questions.

#### Sleep Hygiene Index (SHI)

The SHI is a validated instrument which explores the existence of behaviours and environmental conditions that may compromise an individual’s sleep hygiene or healthy sleep practices. This instrument contains 13 self-rated questions with response options in a 5-point Likert-type scale, ranging from “never” to “always”. The total SHI score ranges from 0 to 52, with higher scores indicating poor sleep hygiene.^41^ A cut-off score of 16 was reported to be appropriate for identifying poor sleep quality among university students with a sensitivity of 77% and specificity of 47.5%.^42^ This indicates that the SHI is a good instrument for detecting individuals with poor sleep hygiene. The SHI was translated to Arabic by our research team using a standard linguistic validation and cultural adaptation process, and has been published elsewhere. The Arabic SHI has moderate internal consistency reliability with a Cronbach’s alpha of 0.589.^43^,^44^

### Data Analysis

The SPSS software version 26 (IBM SPSS^®^ Statistics for Windows; IBM Corp, Armonk, New York, USA) was used for the data analyses. Descriptive statistics were used for analysis of the demographic characteristics and sleeping patterns. The total scores for both PSQI and SHI were computed for each respondent and presented as mean±standard deviation (SD). Mann–Whitney U and Kruskal–Wallis tests were used to measure the differences in global PSQI and SHI scores for different sociodemographic variables. Pearson’s and Spearman’s Rank correlation tests were employed to assess the correlation between the demographic variables and total scores of sleep quality and sleep hygiene. Binary univariate and multivariate logistic regression analyses were also used to assess the cause-effect relationship between the students’ sociodemographic factors and PSQI or SHI total scores. Only variables which passed the p value’s cut-off point of <0.2 in the univariate regression analysis progressed to the multiple logistic regression analysis. This p-value was reported to enhance the univariate model’s ability to identify all truly important variables.^45^,^46^

### Ethical Considerations

Approval was received from QU’s Institutional Review Board (reference number: QU-IRB 977-EA/18). The participants provided informed consent before participating in the study. No personal identifiers were collected, and all responses were anonymous. Our study complies with the Declaration of Helsinki.

## Results

### Participants’ Characteristics

Two thousand and sixty-two students responded to the survey with a response rate of 10.5%. As shown in Table 1, the sample consisted mostly of females (85%) and Qatari students (63%) who were in the age category 18–23 (70%). The demographics of the respondents in this study were mostly representative of those of QU students enrolled in the Fall semester of 2018 as the majority of them were females and Qatari citizens (77.3% and 65.6% respectively).^36^ Most of the respondents were from the Colleges of Arts and Sciences (33.3%), Business and Economics (19.3%), and Engineering (15.7%), which is in line with the QU census reporting these colleges as having the highest number of students.

**Table 1:** Sociodemographic Characteristics of Qatar University Student Respondents (N=2062*)

Variable	n (%)
Gender**	
Male	303 (14.7)
Female	1753 (85.3)
Nationality**	
Qatari	1302 (63.3)
Non-Qatari	756 (36.7)
Age category**	
≤ 23 years	1439 (70)
>23 years	616 (30)
Marital status**	
Single	1616 (78.6)
Married	439 (21.4)
College**	
College of Arts and Sciences	683 (33.3)
College of Business and Economics	397 (19.3)
College of Engineering	322 (15.7)
College of Education	221 (10.8)
College of Law	157 (7.6)
College of Sharia and Islamic studies	123 (6)
College of Health Sciences	78 (3.8)
College of Medicine	36 (1.8)
College of Pharmacy	36 (1.8)
Previous use of sleep aids (open time frame)**	
Herbal	137 (6.6)
Non-prescription medicine (OTC)	370 (17.6)
Prescription medicine	84 (4.1)
None	1560 (75.7)
**Use of multiple medications**	
Prescription and herbal	9 (11.4)
Non-prescription and herbal	50 (63.3)
Prescription and non-prescription products	20 (25.3)

**Notes**: *Total number of valid students who responded to the survey questions. **Missing data.

### Sleep Quality and Sleep Hygiene

Total PSQI scores were calculated for 1549 students. The majority of the respondents (69.7%) had PSQI scores of >5 and a mean PSQI score of 7.57±3.03. As summarized in Table 2, in the previous month, at least one-third of the respondents reported short sleep duration lasting >6 hours (32.5%), difficulty in sleep initiation within 30 minutes of going to bed 3 times or more per week (35.6%), and waking up in the middle of the night or early morning at least 3 times a week (38.2%).

**Table 2:** Students’ Perceptions of Their Sleep Quality (N=2062*)

	n (%)			
**Duration of time required to fall asleep**				
	≤ 15 minutes	16–30 minutes	31-60 Minutes	> 60 minutes
	631 (35.4)	620 (34.8)	313 (17.6)	218 (12.2)
**Average duration of sleep**				
	≥ 7 hours	≥ 6 hours up to < 7 hours	≥ 5 hours up to < 6 hours	< 5 hours
	792 (44.3)	414 (23.2)	330 (18.5)	250 (14.0)
**Sleep patterns**				
	< once a week	Once or twice a week	≥ 3 times a week	None
Lack of sleep within 30 minutes	342 (18.7)	446 (24.4)	650 (35.6)	387 (21.2)
Early awakenings	321 (17.6)	440 (24.1)	697 (38.2)	365 (20)
Sleep efficiency**				
	≥ 85%	75% to < 85%	65% to < 75%	< 65%
	1194 (70.2)	276 (16.2)	145 (8.5)	86 (5.1)
**Subjective sleep quality**				
	Very good	Fairly good	Fairly bad	Very bad
	315 (17.3)	938 (51.5)	378 (20.7)	191 (10.5)
**Causes of sleep interruption**	< once a week	Once or twice a week	≥ 3 times a week	None
Bathroom use	486 (26.6)	405 (22.2)	294 (16.1)	639 (35.0)
Difficulty breathing	307 (16.9)	233 (12.8)	143 (7.9)	1134 (62.4)
Snoring or coughing	245 (13.5)	117 (6.5)	87 (4.8)	1360 (75.2)
Feeling too cold	441 (24.2)	465 (25.5)	331 (18.1)	587 (32.2)
Feeling too hot	456 (25.1)	444 (24.5)	208 (11.5)	707 (39.0)
Nightmares	614 (33.7)	329 (18.1)	143 (7.9)	735 (40.4)
Pain	433 (23.8)	287 (15.8)	190 (10.5)	908 (49.9)
**Consumption of sleep aids within the past month (prescribed or “over the counter”)**				
	< once a week	Once or twice a week	≥ 3 times a week	None
	128 (7.0)	88 (4.8)	69 (3.8)	1532 (84.3)
**Difficulty staying awake while doing different activities (eg driving, eating, participating in social activities)**				
	< once a week	Once or twice a week	≥ 3 times a week	None
	484 (26.6)	448 (24.6)	233 (12.8)	653 (35.9)

**Notes**: *Total number of valid students who responded to the survey questions. **Based on Questions 1 and 3 of the Pittsburgh Sleep Quality Index.^39^

The majority of the students perceived their sleep quality as being good (68.8%) with a sleep efficiency of ≥85% in the previous month (70.2%). Responses revealed that the majority of students identified to have good sleep quality as per the PSQI were aware that their sleep quality was good. On the other hand, only 43% of students with poor sleep quality realized that their sleep quality was poor.

About one-third of the responding students (37.4%) reported experiencing difficulty staying awake while driving, eating meals, or engaging in social activities at least once per week. Moreover, a significant proportion of the students (46.8%) reported a significant difficulty with maintaining their enthusiasm while completing their duties within the previous month. Approximately 25% of the respondents reported previous use of sleep aids, with 15.6% of them indicating that the use was within the past month. Non-prescription products appeared to be the most frequently consumed sleep aids among the respondents.

The majority of the students (78.8%) had SHI scores >16, and a mean score of 21.79±6.69. As shown in Table 3, the most reported negative sleep habits included engaging in awakening activities before sleeping (64.7%) and using bed for things other than sleep, such as watching television and studying (46.4%).

**Table 3:** Frequency of Different Sleep Hygiene Practices Among Qatar University Students (N= 1726*)

Questions	Never n (%)	Rarely n (%)	Sometimes n (%)	Frequently n (%)	Always n (%)
1) I take daytime naps lasting 2 or more hours.	403 (23.3)	503 (29.1)	386 (22.4)	258 (14.9)	176 (10.2)
2) Going to bed at various times from day to day.	120 (7.0)	362 (21.0)	546 (31.6)	429 (24.9)	269 (15.6)
3) Getting out of bed at various times from day to day.	153 (8.9)	476 (27.7)	544 (31.7)	362 (21.1)	182 (10.6)
4) I exercise to the point of sweating within 1 hour of going to bed.	1245 (72.1)	288 (16.7)	125 (7.2)	51 (3.0)	17 (1.0)
5) I stay in bed longer than I should (after waking up) two or three times a week.	243 (14.1)	422 (24.5)	470 (27.3)	353 (20.5)	236 (13.7)
6) I use tobacco or caffeine (for example: coffee or tea) within 4hrs of going to bed or after going to bed.	695 (40.4)	320 (18.6)	360 (20.9)	199 (11.6)	147 (8.5)
7) I do something that may wake me up before bedtime (for example: play video games, use the internet, or clean).	125 (7.3)	146 (8.5)	336 (19.5)	464 (27.0)	649 (37.7)
8) I go to bed feeling stressed, angry, upset, or nervous.	272 (15.8)	463 (26.9)	572 (33.2)	282 (16.4)	132 (7.7)
9) I use my bed for things other than sleeping or marital intimacy (for example: watch television, read, eat, or study).	283 (16.5)	252 (14.7)	388 (22.6)	343 (20.0)	453 (26.4)
10) I sleep on an uncomfortable bed (for example: poor mattress or pillow, too much or not enough blankets).	1162 (67.6)	294 (17.1)	169 (9.8)	60 (3.5)	34 (2.0)
11) I sleep in an uncomfortable bedroom (for example: too bright, too stuffy, too hot, too cold, or too noisy).	1103 (64.2)	319 (18.6)	181 (10.5)	74 (4.3)	42 (2.4)
12) I do important work before bedtime (for example: pay bills, schedule, or study).	261 (15.2)	325 (18.9)	528 (30.8)	364 (21.2)	239 (13.9)
13) I think, plan, or worry when I am in bed.	140 (8.1)	280 (16.3)	520 (30.2)	365 (21.2)	415 (24.1)

**Note**: *Number of participants who have answered at least one of the Sleep Hygiene Index questions.

### Differences in PSQI and SHI Scores

**Figure 1:**
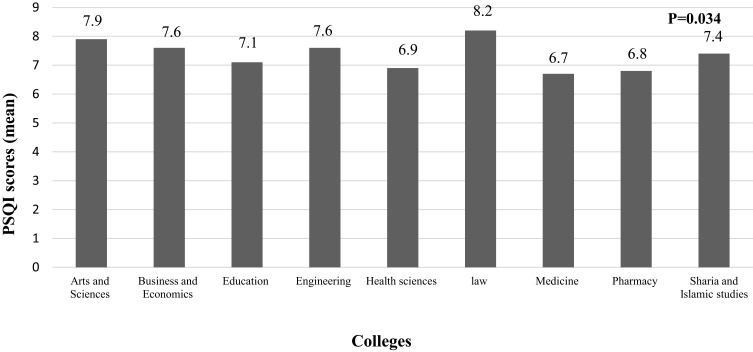
Difference in PSQI scores between different colleges.

**Note**: Bold numbers indicate significant p value.

**Figure 2:**
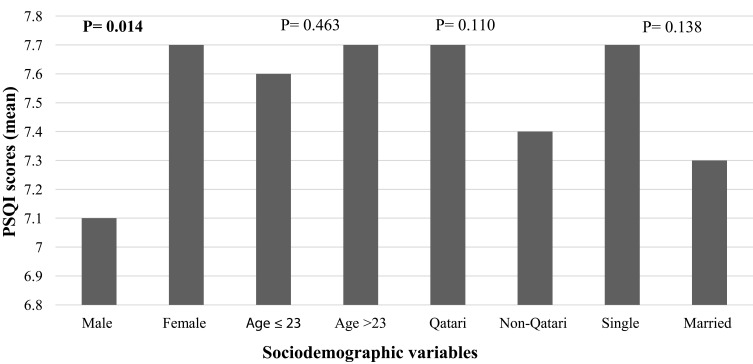
Difference in PSQI scores between sociodemographic variables’ categories.

**Note**: Bold numbers indicate significant p value.

**Figure 3:**
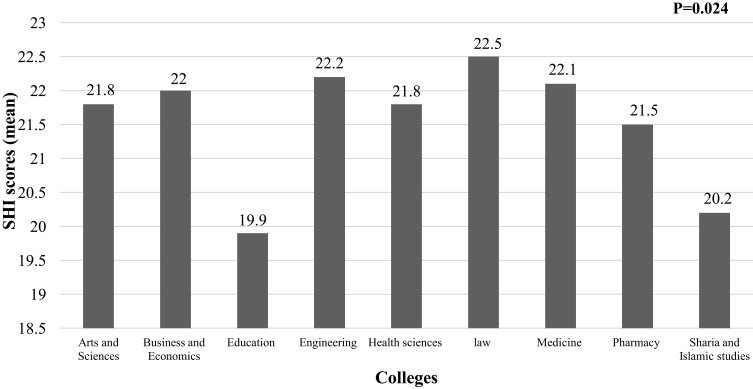
Difference in sleep hygiene scores between colleges.

**Note**: Bold numbers indicate significant p value.

**Figure 4:**
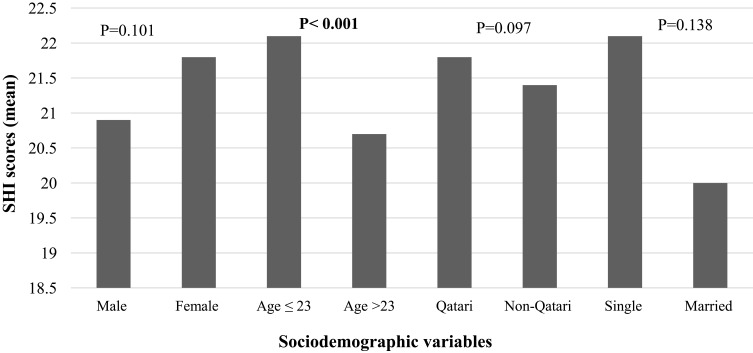
Difference in sleep hygiene scores between different sociodemographic categories.

**Note**: Bold numbers indicate significant p value.

As illustrated in Figures 1–4, the findings revealed a statistically significant difference in sleep quality between different colleges (Figure 1) and gender categories (Figure 2). The worst sleep quality was detected among College of Law students, while the best was reported by students at the College of Medicine. The statistically significant difference between colleges was analysed through a post-hoc pairwise comparison of sleep quality scores, which revealed that this result was driven by the significant differences between each of the following colleges: Medicine, Pharmacy, Education, Health Sciences and Sharia & Islamic Studies with the College of Law (p=0.001, p=0.032, p=0.018, p=0.035 and p=0.037, respectively), and the difference between the College of Education with each of following colleges: Business & Economics and Arts & Sciences (p=0.044 and p=0.013, respectively). Similarly, there were statistically significant differences in the SHI scores between students from different colleges and age categories (p=0.024 and <0.001, respectively) (Figures 3 and 4). The best sleep hygiene (lowest mean SHI scores) was identified among students from the colleges of Education and Sharia & Islamic studies.

### Correlation Between Sleep Quality, Sociodemographic Variables, and Sleep Hygiene

Correlation analysis showed a significant positive correlation between the PSQI and the SHI scores (R=0.394, p<0.001). As detailed in Table 4, gender was the only demographic factor shown to be significantly associated with PSQI scores (p=0.014). While SHI scores were significantly correlated with students’ age and marital status (p<0.001 for both). The use of almost all forms of sleeping aids was also significantly associated with both, the PSQI and SHI scores, except for the use of herbal medicines, which was significantly correlated only with the PSQI (p=<0.001). A significant correlation was found between sleep parameters including sleep latency and efficiency with the total PSQI and SHI scores (p<0.001).

**Table 4:** Sociodemographic Variables Association with Sleep Quality and Sleep Hygiene

Variable	PSQI Global Scores^a^		SHI Total Scores^b^	
	R	*p*-value*	R	*p*-value*
**College**	−0.027	0.291	−0.012	0.614
**Gender**	0.062	**0.014**	0.040	0.101
**Age**	0.019	0.464	−0.098	**< 0.001**
**Marital status**	−0.038	0.138	−0.137	**< 0.001**
**Country of origin**	−0.041	0.110	−0.040	0.097
**Use of herbal products**	0.155	**< 0.001**	0.021	0.383
**Use of OTC products^c^**	0.260	**< 0.001**	0.153	**< 0.001**
**Use of Prescription medicine**	0.120	**< 0.001**	0.061	**0.012**

**Notes**: *p value was calculated using Spearman Rank test. Bold number highlight statistically significant p values.

**Abbreviations**: ^a^PSQI, Pittsburgh Sleep Quality Index; ^b^SHI, Sleep Hygiene Index; ^c^OTC, Over the counter.

### Predictors of Sleep Quality

The findings resulting from the univariate binary logistic regression analysis (Table 5) indicate that the students’ PSQI scores were significantly influenced by the college the respondent belongs to (p=0.031); with the cut-off p value being 0.2. When comparing the PSQI scores of students at the College of Arts & Sciences (CAS) (which had the highest representation in the sample), students at the College of Education had an increased probability of having poor sleep (OR=0.594; 95% CI=0.41–0.861, p=0.006). In comparison to students from the College of Law, students from CAS were 1.4 times more likely to have good sleep quality (OR=1.415, 95% CI=0.884 −2.264, p=0.148). On the other hand, students from CAS were 73% less likely to have good sleep quality when compared to students from the College of Sharia & Islamic Studies (OR=0.733, 95% CI=0.461–1.164, p=0.188). Overall, the contribution of college designation to sleep quality changes was negligible (1%).

**Table 5:** Univariate and Multivariate Logistic Regression for the Association Between Students’ Sociodemographic Characteristics and Sleep Hygiene with Sleep Quality

Variable	Univariate Analysis^a^				Multivariate Analysis^b^			
	B	S.E.	OR	P*	B	S.E.	OR	P**
College***	R2=1.1%				R^2^=7.5%			
**College**				**0.031**				0.357
Business and Economics	0.133	0.163	1.142	0.416	0.101	0.178	0.781	0.571
Education	−0.521	0.189	0.594	**0.006**	−0.370	0.207	0.691	0.075
Engineering	0.010	0.169	1.010	0.953	−0.031	0.187	0.969	0.869
Health sciences	−0.137	0.289	0.872	0.635	−0.156	0.308	0.855	0.613
Law	0.347	0.240	1.415	**0.148**	0.213	0.254	1.237	0.403
Medicine	−0.186	0.420	0.831	0.658	−0.482	0.435	0.617	0.268
Pharmacy	−0.444	0.399	0.642	0.267	−0.550	0.416	0.577	0.185
Sharia and Islamic studies	−0.311	0.236	0.733	**0.188**	−0.177	0.258	0.837	0.492
**Gender**	R2= 0.2%				**0.244**	0.177	1.276	0.167
Female	0.270	0.156	1.31	0.083				
**Age**	R2= 0.1%							
More than 23 years old	−0.125	0.119	0.882	0.292				
**Marital status**	R2= 0.3%				−0.198	0.146	0.821	0.176
Married	−0.304	0.131	0.738	0.020				
**Nationality**	R2 < 0.001%							
Non-Qatari	−0.061	0.115	0.941	0.595				
**Sleep Hygiene**	R2 = 6.7%				1.298	0.137	3.661	<0.001
SHI scores > 16	1.342	0.134	3.825	<0.001				

**Notes**: ^a^Simple analysis was conducted using binary logistic regression to assess the effects of each variable on sleep quality. ^b^Multiple logistic regression conducted to assesses the effect of each variable when other variables are kept constant. *Significant if P value <0.2, **Significant if P value< 0.05, ***College of Arts and sciences used as the reference. Bold numbers highlight statistically significant p values.

Sleep quality was also influenced by the marital status as single students were found to be 74% less likely to have good sleep quality (OR=0.738, 95% CI= 0.571–0.953, p=0.02). However, the impact of this variable on sleep quality was minimal with only 1% of sleep quality’s variability being attributed to marital status. Similarly, gender had a significant effect on sleep quality with male students being more likely to have better sleep quality than female students by 1.3 folds (OR=1.31, 95% CI=0.965–1.78, p=0.083). Additionally, 7% of the change in students’ sleep quality was driven by sleep hygiene as it was found that students with SHI scores of ≤16 were four times more likely to have good sleep quality than their colleagues who scored >16 (OR=3.825, 95% CI=0.965–1.78, p<0.001).

The combined effects of different sociodemographic variables, sleep hygiene, and the college designation explains up to 8% of the variability in sleep quality (R^2^ = 7.5%) (Table 5). Nevertheless, when all factors were taken into consideration in the multiple regression analysis, the effects of all variables except sleep hygiene diminished. Sleep hygiene was shown to have significant influence on sleep quality as students with better sleep hygiene had higher probability of having good sleep quality by approximately 4 folds (OR=3.66, 95% CI=2.8–4.8, p<0.001).

## Discussion

The findings of this study have not only confirmed that poor sleep quality is highly prevalent among QU students, but have also advanced our understanding on sleep hygiene practices, an issue not sufficiently explored in the Arab world and that may be contributory to the high prevalence of poor sleep quality reported among university students. Approximately 70% of the students had poor sleep quality in this study, an incidence that does not appear to correlate with the much lower insomnia prevalence (5.5%) that was found among adults in Qatar attending primary care services.^34^ This discrepancy can be partially explained by the differences in the populations studied and the instruments used in the assessment of sleep. Nevertheless, it is also indicative that despite the high prevalence of poor sleep quality found in our study population, university student do not tend to seek medical care and, thus, the lower insomnia prevalence rates reported in other populations studied may be underestimated.

Inadequate sleep hygiene was the most influential factor impacting sleep quality, reported by 80% of the students in our study. Irregular sleeping schedules, engaging in awakening activities around bedtime, and using the bed for purposes other than sleep (eg, watching television or studying) were the most commonly reported inadequate sleep hygiene practices among the students in our study. There was also a significant correlation between sleep hygiene and sleep quality. The influence of sleep hygiene on sleep quality persisted in the multiple regression analysis, such as students with good sleep hygiene practices were four times more likely to have good sleep quality compared to those with poor sleep hygiene. Similar findings have been reported in other studies.^18^,^23^,^42^,^47^,^48^ One study found that a one-unit increase in sleep hygiene practice score is associated with a corresponding decrease in sleep quality score by 0.08.^18^

Other contributing factors to poor sleep quality were also reported, at rates that are consistent with those reported in similar studies, which may explain the higher risk for insomnia that has been consistently reported among university students.^2^,^13^,^16^,^49^,^50^ Almost half of the students in our study reported going to sleep while feeling angry, stressed, or nervous. Worries about academic performance, time constraints, and future plans are the most commonly reported sources of stress among university students.^51^,^52^ Interestingly, only 25% of the students in our study reported taking regular daytime naps lasting more than 2 hours. Other studies have shown much higher napping rates among university students.^10^,^20^,^25^,^31^ It is possible that the lower rate reported by our study population is because shorter naps of less than 2 hours were not considered as naps in the SHI. As midday napping is a common practice in Arab and Muslim societies, it is possible that this habit may have been underestimated. Cultural adaptation of the SHI to exclude the lower end limit for daytime nap duration of 2 hours is recommended for future studies in Arab populations.

Despite the high prevalence of poor sleep quality reported, the majority of the students in our study perceived their sleep as being good and reported a sleep efficiency of ≥85% in the previous month. Similar findings have been reported in other studies among university students.^8^,^15^,^49^,^53^,^54^ These findings could suggest lack of awareness about the characteristics of a healthy sleep among students, underestimation of the importance of these symptoms, or fear of the stigma associated with sleep complaints since these are usually linked with psychiatric problems.^55^ These findings highlight the importance of using validated objective instruments for sleep quality assessment rather than relying on participants’ self-reports or complaints. Also consistent with other studies, only 25% of students in our study reported using sleeping aids, which suggests that university students experiencing sleeping problems report minimal use of medications to manage their sleeping problem.^15^,^17^,^31^,^50^

Our findings have also revealed a significant difference in sleep quality between students from different colleges with the best sleep quality being recorded among students from the College of Medicine (mean PSQI score of 6.7); a result which contradicts previous literature findings in which medical students were found to have the worst sleep quality.^19^,^56^ In accordance with previous studies, female students in our research had worse sleep quality (mean PSQI score of 7.7).^8^,^24^,^49^ Likewise, females were shown to be more likely to develop insomnia by 1.41 folds in a meta-analysis of 31 studies.^57^

Despite the lack of agreement on the effects of marital status on sleep quality in the literature,^12^,^27^,^47^,^50^ in our study married students appeared to have better sleep quality. Older students (>23 years) were also shown to have better sleep hygiene scores. It has been suggested that these findings could be related to a higher awareness level about sleep hygiene among married and older individuals.^58^ Overall, the literature has conflicting results with regard to the effects of age on sleep quality.^8^,^50^

Although these findings are mostly in line with those reported in similar studies in this population, they highlight the significant influence of sleep hygiene on sleep quality. The high prevalence of inadequate sleep hygiene practices found in this study point to inadequate awareness, and is a call for implementing strategies within universities to improve sleep education throughout the students’ academic experience. The main limitations of this study include not assessing students’ knowledge and beliefs about sleep and sleep hygiene, missing to stratify students according to the year of study and not using proportionate sampling technique. However, the universal sampling method used produced a representative sample (in terms of proportion of students per college).

## Conclusion

This study is the first in Qatar to explore sleep quality and sleep hygiene practices among university students. Poor sleep quality and inadequate sleep hygiene practices were highly prevalent in this population. After accounting for all other variables, sleep hygiene’s negative impact on sleep quality persisted, such that students with good sleep hygiene practices were four times more likely to have good sleep quality. There is a high need to implement strategies within higher educational institutions to increase awareness on sleep hygiene in order to improve sleep quality among university students to ensure academic success.
